# The Effect of Green Exercise on Blood Pressure, Heart Rate and Mood State in Primary School Children

**DOI:** 10.3390/ijerph110403678

**Published:** 2014-04-02

**Authors:** Michael J. Duncan, Neil D. Clarke, Samantha L. Birch, Jason Tallis, Joanne Hankey, Elizabeth Bryant, Emma L. J. Eyre

**Affiliations:** Sport and Exercise Applied Research Group, Faculty of Health and Life Sciences, Coventry University, Coventry CV1 5HB, UK; E-Mails: n.clarke@coventry.ac.uk (N.D.C.); s.birch@coventry.ac.uk (S.L.B.); j.tallis@coventry.ac.uk (J.T.); j.hankey@coventry.ac.uk (J.H.); bryante@uni.coventry.ac.uk (E.B.); eyree@uni.coventry.ac.uk (E.L.J.E.)

**Keywords:** environment, nature, hypotension, cycling

## Abstract

The aim of this study was exploratory and sought to examine the effect on blood pressure (BP), heart rate (HR) and mood state responses in primary school children of moderate intensity cycling whilst viewing a green environment compared to exercise alone. Following ethics approval and parental informed consent, 14 children (seven boys, seven girls, Mean age ± SD = 10 ± 1 years) undertook two, 15 min bouts of cycling at a moderate exercise intensity in a counterbalanced order. In one bout they cycled whilst viewing a film of cycling in a forest setting. In the other condition participants cycled with no visual stimulus. Pre-, immediately post-exercise and 15 min post-exercise, BP, HR and Mood state were assessed. Analysis of variance, indicated significant condition X time interaction for SBP (*p* = 0.04). Bonferroni *post-hoc* pairwise comparisons indicated that systolic blood pressure (SBP) 15 min post exercise was significantly lower following green exercise compared to the control condition (*p* = 0.01). There were no significant differences in diastolic blood pressure (DBP) (all *p* > 0.05). HR immediately post exercise was significantly higher than HR pre exercise irrespective of green exercise or control condition (*p* = 0.001). Mood scores for fatigue were significantly higher and scores for vigor lower 15 min post exercise irrespective of green exercise or control condition (both *p* = 0.0001). Gender was not significant in any analyses (*p* > 0.05). Thus, the present study identifies an augmented post exercise hypotensive effect for children following green exercise compared to exercise alone.

## 1. Introduction

Increasing physical activity levels is one key strategy that has been advocated as a means to curb the alarming increases witnessed over the last 30 years in childhood and adolescent obesity and a range of preventable and lifestyle related diseases in this young population [1]. Although the benefits of engaging in regular physical activity are well publicised, the majority of children do not engage in sufficient physical activity for health benefit [2,3] with approximately 23%–34% of males and 35%–53% and of females aged 11–15 years failing to meet the daily recommendation of 60 min of moderate to vigorous physical activity. Evidence, from longitudinal and cross sectional studies respectively, also suggests that physically active children are more likely to be physically active in adulthood [4] and overweight/obese children are more likely to be overweight/obese adults [5]. 

As a result there is a need to develop activities that can be used to increase physical activity and promote healthy weight in children and adolescents [6,7]. “Green exercise” is one form of intervention which may offer multiple health benefits for those who participate in it [8,9] and may be particularly attractive to children. This includes increasing habitual physical activity, positively impacting perceived stress [10] and improving well being (e.g., self-esteem, mood) [8,9]. 

Evidence from experimental studies indicates that simply viewing scenes from nature can have an immunising effect on long term stress and well-being (e.g., [11]), improve mood [12,13,14] and improve attention [15]. Recent research has also shown positive changes in autonomic control of the heart following 5 min passive viewing of images of the natural environment [16]. Pretty *et al*. [9] suggested that there may be a synergistic effect in undertaking physical activity whilst being exposed to nature and termed this “Green Exercise” based on earlier observations by Hayashi *et al*. [17]. This premise is attractive in terms of potential public health applications although few studies have presented robust data linking both exercise and the green environment synergistically. One study by Pretty *et al*. [9] reported significant reductions in systolic (SBP) and diastolic (DBP) blood pressure in adults following 20 min of treadmill walking/jogging at a “fairly light” intensity whilst viewing still images of the natural/green environment compared to exercise alone. The findings presented by Pretty *et al*. [9] are interesting and potentially useful for public health. Given data suggesting that hypertension is a chronic health problem worldwide [18] and the fact that many preventive programmes prescribe exercise as a non pharmacological means by which to positively change blood pressure status [19], understanding the role that exercise plays in BP may be important for effective exercise prescription. Recent longitudinal research has also highlighted that physical activity outdoors is related to lower BP values in children [20] and that regular physical activity is associated with higher health related quality of life [21]. If exercising whilst viewing scenes of nature augments the hypotensive effect seen post exercise, it may be a useful consideration for public health practitioners in prescribing exercise to reduce health risk. 

However, while there are data suggesting that green exercise results in improvements in health and well being, the majority of studies to date have been conducted in adult populations and have tended to examine “light” intensity exercise. However, a dose response relationship for exercise intensity in green exercise has been identified [22]. Recent research by Reed *et al*. [23] has also focused on the impact of green exercise on children’s self-esteem. In their study, Reed *et al*. [23] asked 75, 11–12-year olds to complete two 1.5 km runs; one in a natural green environment and one in an urban environment. The results of this study suggested no significant effect of green exercise on self-esteem, rating of perceived exertion or exercise enjoyment over exercise in an urban environment. Reed *et al*. [23] noted that these results were contrary to data in adult based samples and also suggested that green exercise might be used as a tool to engage children in moderate to vigorous physical activity, particularly for those who are less habitually active. Other recent work examined the effect of two, 15 min bouts of exercise at 50% heart rate reserve on self-esteem and mood state in 25 adolescents [24]. In one condition participants viewed scenes of natural environments and in the other scenes of built environments. However, although there were significant effects for exercise on mood and self-esteem, there was no effect of viewing different scenes on these responses in this adolescent population [24].

Consequently, there is a need to investigate the benefits of introducing green exercise for different populations and using different types, durations and intensities of exercise [9]. Therefore, the present study was exploratory in nature and sought to build on prior research by examining the effects of moderate intensity cycling whilst viewing a green environment compared to exercise alone on blood pressure (BP), heart rate (HR) and mood state responses in primary school children. 

## 2. Methods

### 2.1. Participants

Following ethics approval and parental informed consent, 14 children (seven boys, seven girls, Mean age ± SD = 10 ± 1 years) agreed to participate. Children were selected from school year 5 (ages 9–10) in one primary school in Coventry, UK. Participants who provided signed parental informed consent forms and assented to participate were included within the sample for this exploratory study. Seven (50%, three boys, four girls) of the sample were classified as normal weight and seven (50%, four boys, three girls) as overweight/obese according to IOTF cutpoints [25]. Mean ± SD of body mass index was 19.2 ± 2.9 kg/m^2^.

### 2.2. Procedures

In order to examine the effect of green exercise *vs.* exercise alone on blood pressure, heart rate and mood state, each participant completed 2 trials, each separated by at least 24 h in a counterbalanced order. In one trial participants cycled (Lode Corival Pediatric, Lode, Groningen, The Netherlands) for 15 min at a moderate intensity (Control). In the control condition, participants cycled whilst viewing a blank screen. In the other trial, participants cycled for 15 min whilst watching a film of cycling in a forest environment (Through the Forest; World Nature Video, Lunteren, The Netherlands). The film was projected onto a 70 cm (wide) × 100 cm (high) screen with participants being seated on a bike 100 cm away from the screen. The cycling speed during the film was set at 8 km/h which was equivalent to the speed obtained by children whilst cycling in pilot work. Visual stimulation in the room where the data collection took place was kept to a minimum. Walls were blank and there were no visible windows from which the participant could look out from. Investigators sat behind the participants on the cycle ergometer so they could not be viewed during the exercise protocol. Each participant was requested to maintain a cycling RPM of between70–80 rpm during the trials.

Prior to all trials resting heart rate was determined. Resting heart rate (HRrest) was also obtained from each participant by getting them to lie down in a prone position for 10 min whilst wearing a heart rate monitor (Polar RS400, Polar Electro Oy, Kempele, Finland), in a quiet room void of visual or auditory distractions. Maximum heart rate (HRmax) was estimated at 220 minus the participant’s age. Both HRrest and HRmax were then recorded and used to calculate 50% heart rate reserve (HRR) [26] to be used in subsequent exercise trials. The target heart rate of 50%HRR was employed as prior research has identified this as moderate intensity for children [27] with moderate intensity exercise being recommended as important for health benefit [3,27].

Pre-, immediately post- and 15 min post-exercise each participant completed measures of resting BP and HR. Pre- and 15 min post-exercise each participant completed measures of mood state. In the 15 min period post exercise, participants sat alone in the same room where the exercise had taken place in order to minimize any distractions which might have influenced the post-exercise BP and mood response. Prior to any data collection, participants were familiarized with the equipment to be used. There was also only 15 min rest before baseline measurement and the 15 min post exercise measurement.

### 2.3. Assessment of Blood Pressure and Heart Rate

Blood pressure was assessed by trained researchers in the school setting. Children were assessed in a seated position, with feet flat on the floor on an individual basis and following a 10–15 min rest period. Measurements were taken in the morning using an automated oscillometric device (Omron HEM907XL; Omron Healthcare Europe BV, Hoofddorp, The Netherlands). The BP cuff was applied to the right arm with the lower margin of the cuff approximately 2 cm above the elbow crease and with the arrow on the cuff aligned with the brachial artery. The cuff was wrapped to a tightness allowing two fingers to be inserted under the top and bottom of the cuff. Cuff size used was determined based on mid-upper arm circumference. Two consecutive BP measurements were taken. The mean of these two measurements was used for analysis. If the two measurements differed by 2 mmHg or more the protocol was repeated (two new measurements until the difference did not exceed 2 mmHg). Assessment of BP followed recommended guidelines for determination of resting BP in pediatric populations [28]. This method of BP assessment has also been found to be reliable in pediatric populations [29]. Heart rate was determined prior to BP assessment using a heart rate monitor (Polar RS400, Polar Electro Oy, Kempele, Finland).

### 2.4. Assessment of Mood State

Mood state was assessed using the fatigue, tension and vigor subscales of the Brunel Mood State Inventory (BRUMS), [30]. This self report measure is well established as a reliable and valid measure of mood state [30,31] and has been used in adolescents and children as young as 6 years of age [32,33]. The fatigue and vigor subscales were specifically chosen as they were considered more appropriate in the context of exercise responses in children compared to the non selected BRUMS subscales (confusion; anger; depression). The separate subscales within the BRUMS are considered independent of each other and although can be interrelated, the BRUMS can be used to provide a holistic mood measure or as an indication of separate individual aspects of mood [34]. Mood state assessment was undertaken pre exercise and 15minutes post exercise and was completed before measures of heart rate or blood pressure. Raw scores were converted to T-scores for subsequent analysis as recommended [30]. Although the BRUMS has been used with children as young as 6 [33], it has not been formally validated with children under 12 years of age. As a consequence, prior to commencing the experimental protocol, the mood state questionnaire was fully explained to the children and the researchers explained the meaning of each of the adjectives in the questionnaire to the children. In this way, in the absence of a gold standard to assess mood in children, we sought to obtain as reliable and robust mood state data as possible using an established scale that has shown validity and reliability in 12-year olds.

### 2.5. Statistical Analysis

Data were analysed in a number of ways. Paired samples t-tests were used to examine any differences at baseline between green exercise and control conditions. A priori analysis of baseline data, using independent t-tests, indicated no significant differences due to gender (*p* > 0.05 in all cases) with gender being subsequently removed from additional analysis. A series of 2 (condition) × 3 (time) ways repeated measures ANOVAs were employed to examine any changes in SBP, DBP and HR. Any changes in mood state were assessed using a series of 2 (condition) × 2 (time) ways repeated measures multivariate analysis of variance (MANOVA). Where significant differences were found, Bonferroni *post-hoc* pairwise comparisons were used to determine where the differences lay. Partial eta squared (η^2^) was also used as a measure of effect size. IBM SPSS Statistics for Windows, Version 20.0 (IBM Corp., Armonk, NY, USA) was used for all analysis and statistical significance was set, a priori, at *p* = 0.05. Data are reported as mean ± SE.

## 3. Results

**Figure 1:**
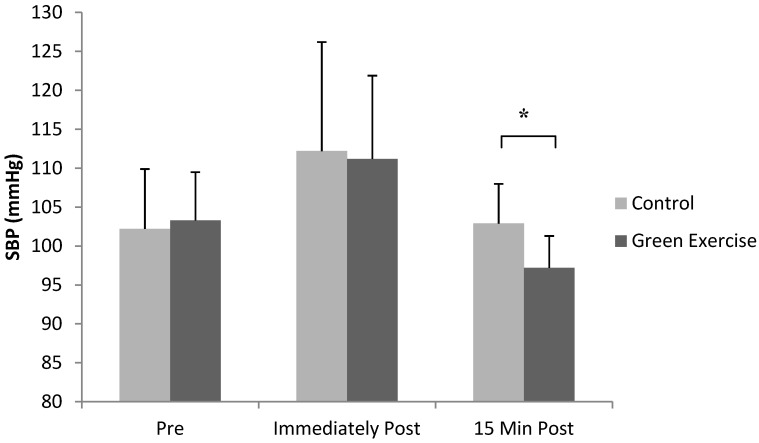
Mean ± SE of SBP scores, pre, immediately post and 15 min post green exercise or exercise alone (n = 14, * *p* = 0.01).

There were no significant differences in any of the assessed variables at baseline (*p* > 0.05 in all cases). Results in regard to BP indicated a significant condition X time interaction for SBP (F (2,26) = 3.49, *p* = 0.04, Pɳ^2^ = 0.212, See Figure 1). Bonferroni *post-hoc* pairwise comparisons indicated that SBP 15 min post exercise was significantly lower following green exercise compared to the control condition (*p* = 0.01). SBP was not significantly different pre- or immediately post-exercise between the green exercise and control conditions. There were no significant main effects for condition or time or interaction between the two for DBP (all *p* > 0.05). 

HR responses did not differ between conditions and there was not a significant condition X time interaction for HR (both *p* > 0.05). There was however a significant main effect for time (F (2,26) = 47.19, *p* = 0.0001, Pɳ^2^ = 0.784). Bonferroni *post-hoc* pairwise comparisons indicate that HR immediately post-exercise was significantly higher than HR pre-exercise (*p* = 0.001) and 15 min post-exercise (*p* = 0.01). Mean ± SE of HR was 82 ± 2 bpm pre-exercise, 104 ± 2 bpm immediately post-exercise and 93 ± 2 bpm 15 min post-exercise. Gender was not significant (*p* > 0.05 in all cases). Mean ± SE for SBP, DBP and HR pre, immediately post and 15 min post-exercise in green exercise and control conditions are presented in Table 1.

**Table 1:** Mean ± (SE) and 95% Confidence Intervals of SBP, DBP and HR pre, immediately post and 15 min post exercise in green exercise and control conditions.

Outcome Variable	Green Exercise (n = 14)						Control (n = 14)					
	Pre		Immediately Post		15 min Post		Pre		Immediately Post		15 min Post	
	M (SE)	95% CI	M (SE)	95% CI	M (SE)	95% CI	M (SE)	95% CI	M (SE)	95% CI	M (SE)	95% CI
SBP (mmHg)	103.9 (2.1)	99.5–108.2	111.2 (2.8)	108.1–117.3	97.2 (1.5)	93.9–100.4	102.2 (1.7)	98.4–105.9	112.7 (4.1)	103–121.4	102.7 (2.2)	99.1–108.6
DBP (mmHg)	69.5 (2.4)	64.2–74.8	68.4 (3.4)	61–75.8	66.6 (1.9)	62.6–70.5	64.7 (1.9)	60.4–68.9	70.6 (3.5)	62.9–78.2	64.6 (2.2)	58.8–68.3
HR (bpm)	81 (2)	76–84	102 (3)	95–108	93 (2)	89–97	83 (3)	76–89	106 (2)	101–109	94 (3)	86–101

Data from repeated measures MANOVA pertaining to mood state indicated a significant mood scale X pre-post interaction (*F* (2,12) = 48.6, *p=* 0.0001, Wilks’ Lambda = 0.11, Pɳ^2^ = 0.89). Bonferroni *post-hoc* comparisons indicated that fatigue scores were significantly greater post-exercise compared to pre- (*p* = 0.001) and vigor scores were significantly lower pre- to post-exercise (*p* = 0.001). Scores for tension were not significantly different (*p* > 0.05). Mean ± SE of T-scores were 39 ± 3.9 and 47.1 ± 5.4 for fatigue and 51.5 ± 5.4 and 43.8 ± 6.6 for vigor pre- and post-exercise respectively. Mean ± SE of T-scores with 95% confidence intervals for BRUMS subscales pre and post green exercise and control conditions are presented in Table 2. Accounting for the possibility that BMI may have influenced the results of the present study, the analyses were rerun covarying for BMI. This did not change the results of the statistical analysis and as subsequently not reported further.

**Table 2:** Mean ± SE and 95% Confidence Intervals for fatigue, vigor and tension mood state scores, pre and 15 min post exercise in green exercise and control conditions.

Outcome Variable	Green Exercise				Control				*p*
	Pre		Post		Pre		Post		
	M (SE)	95% CI	M (SE)	95% CI	M (SE)	95% CI	M (SE)	95% CI	
Fatigue	39.3 (2.2)	39–42	45.3 (2.3)	43.5–49.2	39.8 (1.6)	37.8–42.2	47(2.8)	44.5–50	0.001 *
Vigor	52.1 (2.6)	49–55	45 (2.8)	42.1–49	51.5 (3.1)	48.6–54.6	44.4 (3.0)	40.6–48.6	0.001 *
Tension	45.8 (1.8)	43.1–48	44.4 (1.1)	42.9–46.3	45 (1.3)	42.9–47	44.5 (.8)	43–45.6	>0.05

Note: * pre to post.

## 4. Discussion

The current study was exploratory in nature and extends the current literature examining the health enhancing effects of green exercise by presenting data on the effect of green exercise compared to exercise alone on BP, HR and mood state data in children. The present study identifies an augmented post exercise hypotensive effect for children following green exercise compared to exercise alone. As such these results agree with prior research showing decreases in BP in adults following walking/jogging on a treadmill whilst viewing scenes of nature [9]. The current results also support claims made by previous authors that viewing scenes of nature results in positive cardiovascular responses [16] compared to viewing control scenes. However, the results of the present study in relation to mood state are contrary to adult based studies which have reported positive mood changes following exposure to green scenes in adult samples [9,13,14]. The results of this study do however support recent work by Reed and Wood [23] that showed no effect of green exercise on psychological measures. The discrepancy between the current study and this prior work [9,13,14] is not known although children and adults mood responses do differ [22] and recently Ekkekakis and Petruzzello [35] have suggested that assuming all individuals will respond similarly to a given exercise dose is too simplistic. It may therefore be that the effect of exercise stimulus (with or without scenes of nature) in children differs to that in adults. It is also possible that there are generational differences in connectedness to nature between adults and children that may explain such discrepancies highlighted here. Further research would however be needed to examine this assertion.

The current study has also built on this area by using a moving image of cycling in a forest at the average speed of the cycle ergometer. Arguably this form of simulated green exercise might be more realistic than simply viewing static images of nature and might be more engaging for participants. This latter point is however speculative and requires further investigation. Despite this, consideration should be given to the potential mechanism for the hypotensive effect in the presence of nature reported here. However, the underlying physiological mechanisms involved in response to green exercise are unknown [16]. Prior laboratory studies have suggested that viewing nature alone can have a restorative effect on HR and BP [36,37] after inducing stress and then viewing scenes of nature. These changes have been postulated to be induced by the autonomic nervous system [16]. Recent work by Gladwell *et al*. [14] has also supported this view that changes in autonomic control of the heart promoting increased vagal activity after viewing scenes of nature promote positive changes in other physiological parameters related to health including BP. Park *et al*. [38] have suggested that this change is due to increased relaxation, and subsequent modification of breathing rate and depth, in participants viewing scenes of nature. There have also been suggestions that prior exercise might act synergistically and augment these physiological changes when coupled with viewing scenes of nature [16]. The current study would appear to support this assertion.

Although the results of the present study do suggest a positive health effect of green exercise over exercise alone in children, the present work does have some limitations. The study was exploratory in nature and sought to examine the feasibility of green exercise in primary school children. Such feasibility type studies are needed to establish whether an approach works prior to larger scale research studies. As a consequence, the data presented here lack statistical power and a posteriori power analysis indicated a sample size of 36 children would be required in future studies. Conducting laboratory research with pediatric populations presents different challenges than with adult participants. As a consequence, only a 15 min follow up period was employed post-exercise. Whilst this is similar to other work examining green exercise [9], assessing post-exercise responses for a longer period (e.g., 60 min) would have provided evidence of any sustained post-exercise hypotensive effect in a similar way to prior studies purely examining post-exercise hypotension [39]. Coupling BP and HR assessment with measurement of heart rate variability would also have been useful in explaining the mechanisms by which green exercise influences post exercise BP. It is also difficult to identify whether the hypotensive effect identified here is clinically meaningful. In the present study an electromagnetically braked ergometer was employed enabling the resistance to be modified as a result of fluctuations in cycling cadence during each trial. However, although this was employed to ensure the participants remained at their target HR, we did not assess power output during the trials. In addition, assessing mood pre and only at 15 min post exercise may have meant that any mood enhancing effect of green exercise had dissipated by the time of measurement. Future studies might therefore want to also consider examining mood changes immediately post exercise to account for this possibility. We are also assuming that the changes reported here are as a result of viewing a video depicting green exercise. However, future studies should endeavor to include further video based conditions to better understand whether it is actually the simulated green exercise specifically or simply the distraction of moving images that elicits the effects presented here. Whether viewing other forms of video may elicit similar changes in BP (e.g., music videos) would also be an interesting future study. Moderate intensity exercise was used in the current study, principally due to its recognized importance in enhancing health in children [3,27]. However, examining any interaction between exercise intensity during green exercise may be useful in providing evidence of dose-response effects of this mode of exercise. Other relevant data including parental lifestyle choices, participant’s physical activity levels and dietary habits were not assessed in our sample. Such data may influence exercise responses so needs to be considered in future studies. It is also possible that children from different weight status groups may respond differently to green exercise. Using a larger sample size in future research would be needed to establish if such a difference exists. Finally, the data presented here are acute in nature only and further research is needed which examines the longer term efficacy of simulated green exercise on health parameters in children before recommendations can be made regarding its use in public health programmes.

## 5. Conclusions

This study examined the effects of green exercise compared to exercise alone on BP, HR and mood state data in children. The present study identifies an augmented post-exercise hypotensive effect for children following green exercise compared to exercise alone. 
